# A systemic ultrasound positioning protocol for nasointestinal tube in critically ill patients

**DOI:** 10.1186/s13054-021-03641-2

**Published:** 2021-06-19

**Authors:** Ruizhong Ye, Xuping Cheng, Huihui Chai, Chengzhong Peng, Jingquan Liu, Jiyong Jing

**Affiliations:** 1 Department of Ultrasound Medicine, Zhejiang Provincial People’s Hospital, Affiliated People’s Hospital, Hangzhou Medical College, Hangzhou, 310014 Zhejiang China; 2 Department of Intensive Care, Dongyang People Hospital, Jinhua, 322100 Zhejiang China; 3 Graduate Department, Bengbu Medical College, No. 2600, Donghai Avenue, Bengbu, 233000 Anhui China; 4 Department of Intensive Care, Zhejiang Provincial People’s Hospital, Affiliated People’s Hospital, Hangzhou Medical College, Hangzhou, 310014 Zhejiang China; 5 Department of Medical Education and Simulation Center, Zhejiang Provincial People’s Hospital, Affiliated People’s Hospital, Hangzhou Medical College, Hangzhou, 310014 Zhejiang China

**Dear Editor,**

Critically ill patients have a high nutritional risk for a variety of reasons such as insufficient nutrient intake, and increased nutrient loss. Malnutrition readily impairs organ and immune function, increasing the risk of infection and mortality. Many clinical practice guidelines recommend enteral nutrition (EN) for patients within 24 to 48 h of entering the intensive care unit (ICU) [1, 2]. Patients unsuitable for EN by nasogastric tube, need to be provided with post-pyloric feeding. EN through nasointestinal tube (NIT) is the preferred choice, as it can effectively avoid aspiration caused by reflux, and enhance feeding tolerance. Hence, quick and accurate NIT post-pylorus placement and positioning are crucial [2].

The commonly used methods for aiding placement and positioning of NITs, include abdominal X-ray, auscultation, observation of aspirated fluid, measuring pH, and use of electromagnetic devices and integrated real-time imaging systems. However, these methods have shortcomings, including a lack of visualization, exposure to ionizing radiation, image overlap, or are not readily available, which can lead to subjective placement, low positioning accuracy, and additional costs [3, 4]. Ultrasonography has attracted attention owing to its ready availability, safety, ease of visualization, three-dimensional spatial view, lack of additional cost, and the availability of new techniques such as contrast-enhanced ultrasound [5]. Ultrasonography has been used for rapid positioning of feeding tubes in COVID-19 patients, which reduces the risk of virus transmission [6].

With the new ultrasonic techniques and methods applied in NIT positioning, requirements for the sonographer (e.g., detailed knowledge of anatomy) and the ultrasound equipment (e.g., an ultrasound contrast function) have also increased. The isolated use of each method or technique can necessitate repeated examinations and take increased time. Having a systemic ultrasound positioning method is important for the promotion and application of ultrasonography.

Based on these considerations, we established a systemic ultrasound positioning protocol (Fig. 1) for NIT placement in critically ill patients, based on research as follows [5]: (1) Four critical anatomical parts, the cervical esophagus, pylorus, duodenal bulb, and horizontal part of the duodenum, were determined. Their ultrasound views were standardized. (2) The duodenal bulb was located by identifying the gallbladder and head of the pancreas. The horizontal part of the duodenum was located by identifying the abdominal aorta, inferior vena cava, and mesenteric vessels. The latter was determined as the part for a prioritized examination for its less time-consuming. (3) The number of cross-sections of the NIT in the short-axis view of the pylorus helps to confirm whether it is placed post-pylorus. An odd number indicates an anterior or post-pylorus tube placement, which needs to be considered with the NIT insertion depth. An even number indicates anterior pyloric placement. (4) New acoustic signs of the NIT (Fig. 2) and the use of new techniques effectively improve the imaging effect of the tube. Abdominal X-ray was used as the gold standard in our study of 157 patients. The performance indicators for post-pyloric NIT positioning of this protocol were 96.4%, 90.0%, 98.5%, 78.3%, 95.5%, and 0.81, for the sensitivity, specificity, positive predictive value, negative predictive value, accuracy, and the kappa coefficient, respectively. The median examination time was 20 s [15–33].

**Fig. 1:**
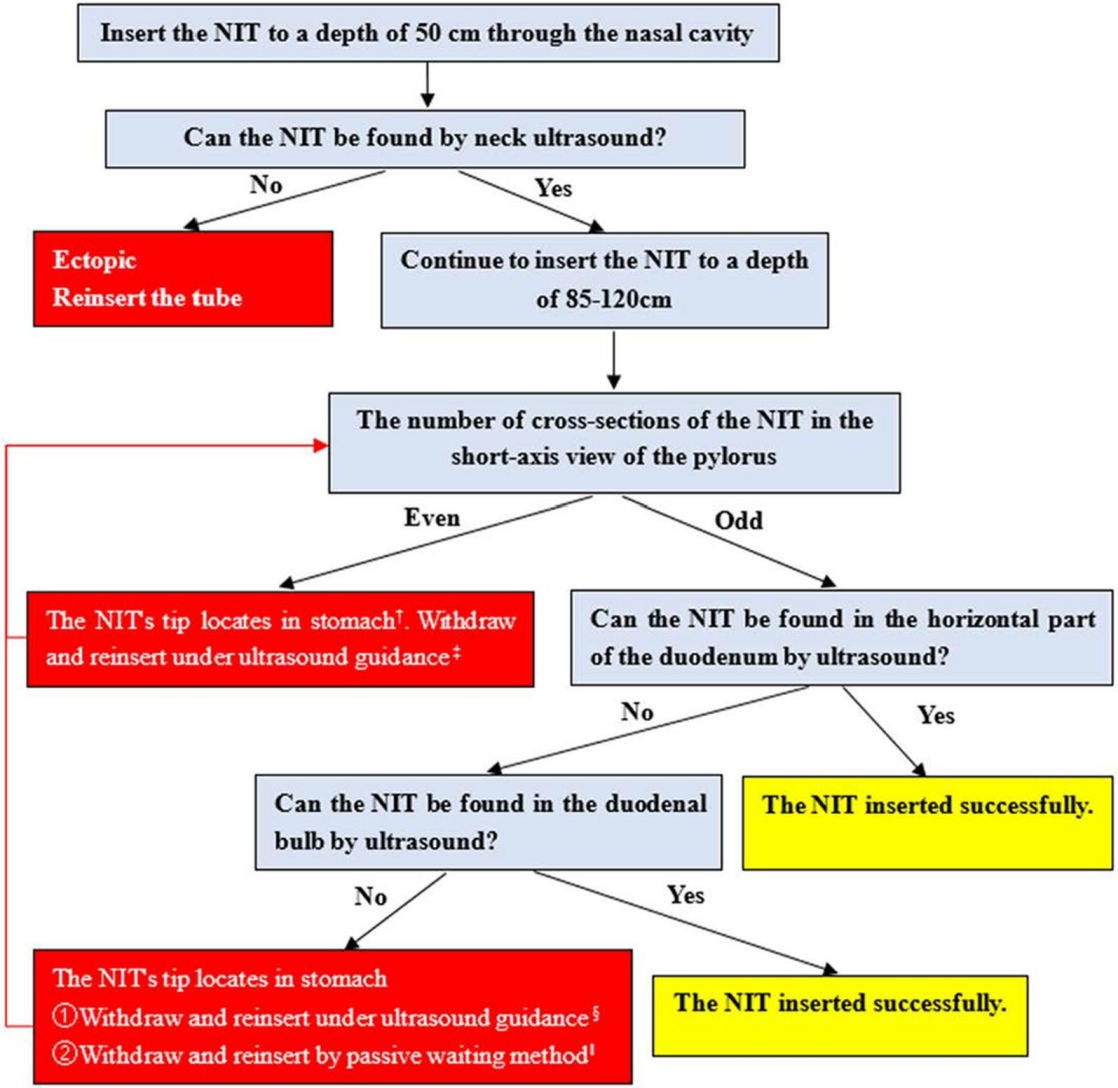
Illustration of the systemic ultrasound protocol for positioning nasointestinal tubes (NITs) in critically ill patients. ^†^There are two situations: (1) The NIT coils in the stomach cavity; (2) The NIT turns back post-pylorus, with the tip locating in the stomach cavity. ^‡^Based on these two situations, different methods are adopted, as follows: (1) When the NIT coils in the stomach cavity, it should be withdrawn to a depth of about 50 cm and then reinserted under ultrasound guidance. (2) When the NIT turns back post-pylorus, it should be withdrawn to a depth of about 75 cm (the tip roughly located in the pylorus) and then reinserted it under ultrasound guidance. ^§^The NIT is withdrawn to a depth of about 50 cm and then reinserted under ultrasound guidance. ^ǁ^If there is a recurrent failure of NIT insertion under ultrasound guidance, adopt a passive waiting method, and allow the NIT to be guided through the pylorus using gastrointestinal peristalsis

**Fig. 2:**
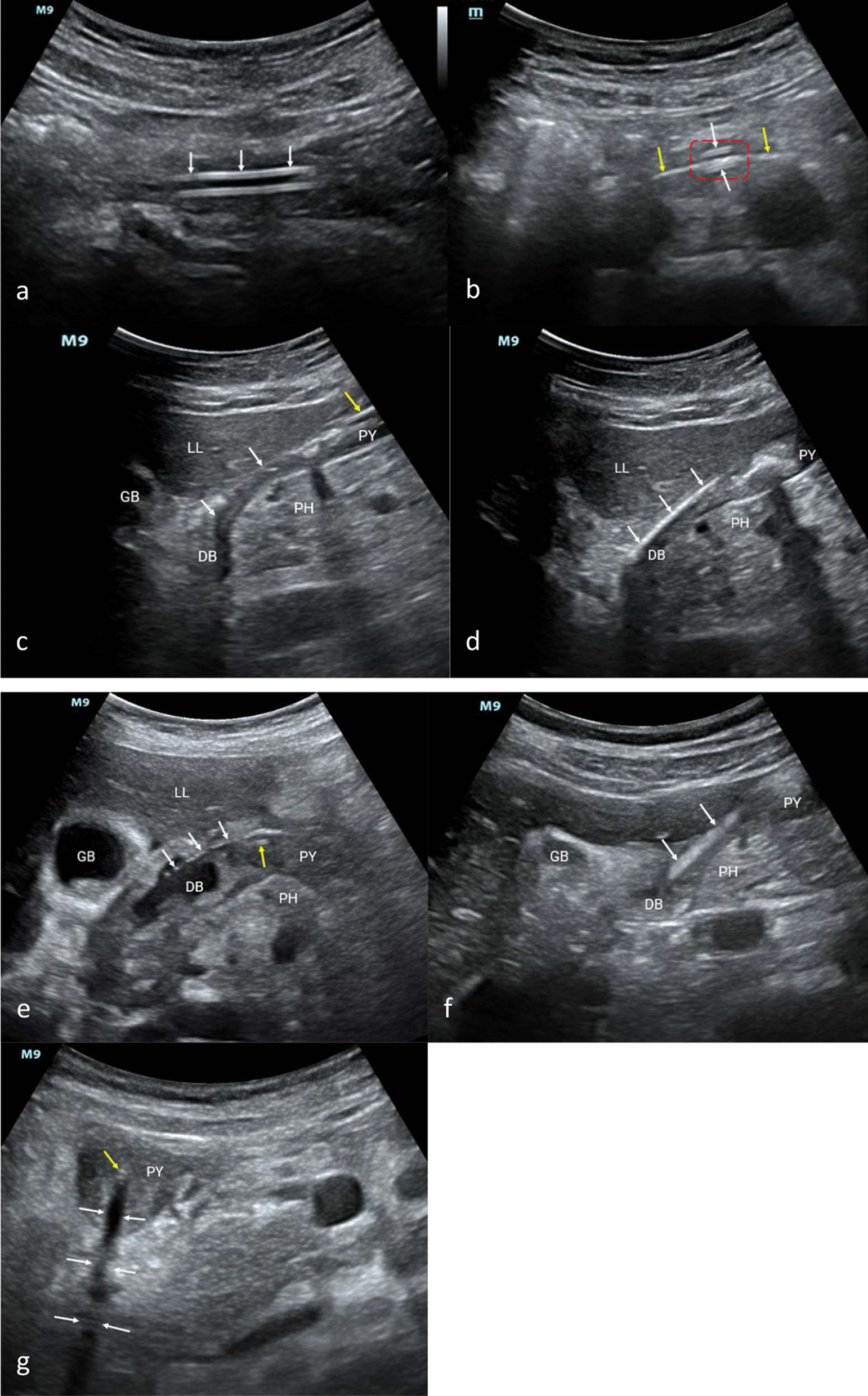
Acoustic signs of the nasointestinal tube (NIT) on ultrasound. **a** Double-track sign: white arrows; **b** Five lines sign: red dotted box; Guidewire: yellow arrows; Wall of the NIT: white arrows; **c** Bar shadow sign: white arrows; NIT: yellow arrow; **d**: Bright band sign: white arrows; **e** Gas bead-like sign: white arrows; NIT: yellow arrow; **f**: Dynamic water flow sign: white arrows; **g**: Short-axis acoustic shadow sign: white arrows. NIT: yellow arrow. DB, duodenal bulb; GB, gallbladder; LL, left liver; PH, pancreatic head; PY, pylorus

NIT positioning can be rapidly and accurately performed using this protocol, helping critically ill patients achieve early EN. There were some limitations in this study. It was a single-center study and patients with abnormal anatomy of the digestive tract(e.g., genetic variation or gastrectomy) were excluded. A multicenter study with a large sample size is required to verify the feasibility of using this protocol. A comparative study on the effect of sonographer proficiency on the accuracy of NIT positioning is also necessary.

## Data Availability

Some or all datasets generated and/or analyzed during the current study are not publicly available but are available from the corresponding author on reasonable request.
